# Application of telemedicine and eHealth technology for clinical services in response to COVID‑19 pandemic

**DOI:** 10.1007/s12553-020-00516-4

**Published:** 2021-01-14

**Authors:** Anthony Jnr. Bokolo

**Affiliations:** Department of Computer Science, Norwegian University of Science and Technology, NTNU, NO-7491 Trondheim, Norway

**Keywords:** Telemedicine, eHealth technology, COVID-19, Coronavirus 2019, Clinical services, Pandemic

## Abstract

Telemedicine and eHealth refer to the use of information and communication technology (ICT) embedded in software programs with highspeed telecommunications systems for delivery, management, and monitoring of healthcare services. Application of telemedicine have become timely while providing great potentials to protect both medical practitioners and patients, as well as limit social mobility of patients contributing to reduce the spread of the virus. This study employs data from the existing literature to describe the application of telemedicine and eHealth as a proactive measure to improve clinical care. Findings from this study present the significance of telemedicine and current applications adopted during the pandemic. More importantly, the findings present practical application of telemedicine and eHealth for clinical services. Also, polices initiated across the world to promote management of COVID-19 are discussed. Respectively, this study suggests that telemedicine and eHealth can be adopted in times of health emergency, as a convenient, safe, scalable, effective, and green method of providing clinical care.

## Introduction

Telemedicine and eHealth platforms which is the use of highspeed telecommunications systems and software application technologies for the provision, management and monitoring of medical-care services possess the potential to protect medical practitioners and outpatients from COVID-19 exposure [1]. These applications can supports patients and physicians to communicate 24/7, using webcam-enabled computers or smartphones [2]. Telemedicine enables patients to consult a physician via teleconferencing, in real-time, seek advice regarding their health problems [3]. Telemedicine and eHealth platforms provide an opportunity of bringing patient and physician together digitally [4], without requiring physical contact relieving congested clinical services and avoids the risk of further infection [5, 6].

Consequently, telemedicine platforms provide a unique and innovative solutions to help address the critical needs of outpatients who may require medical attention but are unable to receive it due to lack of resources or limited access [3]. Indeed, telemedicine is a critical asset, with significant implications across the entire healthcare delivery spectrum which provides several advantages, especially in the setting of routine care and in situations where services may not require direct physician-patient interaction such as in medical consultations [3]. This helps to reduce resource use such as Personal Protective Equipment (PPE), enhance access to healthcare, and at the same time reduces the risk of direct person-to-person transmission of COVID‑19 [5, 7].

Evidently, telemedicine and eHealth platforms are undeniably important for global management of COVID-19 [8], as the pandemic looms over the world [9]. Medical-centers leveraging and adopting telemedicine and eHealth platforms for patient care will gain several returns, including decrease of medical practitioners’ burnout, workforce sustainability, limitation of medical practitioners’ exposure, and decrease of PPE including N95 respirators and surgical masks waste [2, 9]. In the United State (US) more than 50 medical-centers already have such eHealth platforms and are currently adopting telemedicine to support physicians to see outpatients who are at home [2, 10].

Telemedicine and eHealth platforms enable physicians to remotely identify patients who may require further physical care [11]. Although, the use of telemedicine platforms in nations health care system is challenged with issues such as lack of regulatory frameworks that promote a safe and secure adoption of digital medical solutions for healthcare during the COVID-19 pandemic [12]. Also, during the COVID-19 pandemic there are fewer studies that provides guidance and recommendation on how telemedicine and eHealth platforms can be adopted to provide clinical services [13]. According this study describes the significance of telemedicine and eHealth platforms during the pandemic in providing clinical services. The reminder of the paper is structured as section 2 is methodology, section 3 is findings. Section 4 is discussion and implications, and lastly section 5 is conclusion.

## Methodology

This study employs a structured review of secondary sources on the role of telemedicine and eHealth to provide clinical services amid and beyond the COVID-19 pandemic. Existing evidence from journal papers, conference proceedings, and document reports are utilized as seen in the reference section. A search strategy was carried out for studies related to telemedicine and eHealth during COVID-19 pandemic using online libraries during the first week of May 2020 in Google Scholar, PubMed, ScienceDirect, ProQuest, Springer, Sage, Taylor & Francis, IEEE Xplore, Wiley, ACM, Emerald, Inderscience, ISI Web of Science, and Scopus. Additionally, in employing the search specific keywords was used to query online libraries using Boolean OR/AND operators to improve the search relevance. The keywords comprise of “telemedicine”, “eHealth” “telehealth”, “mobile health”, “remote medicine”, “COVID-19”, “corona virus 2019” and “pandemic”.

At the end of the search 98 articles were retrieved. After, checking for duplicates and excluding papers not related to the research questions being reviewed 30 papers were selected. After which 2 papers was included based on snowball cross-referencing resulting to 32 papers. Each of the selected studies are synthesized to provide evidence on how telemedicine and eHealth can be applied to provide clinical services amid the COVID-19 pandemic. Thus, this study aims to address the following research questions;

- **RQ1:** What is the significance of applying telemedicine and eHealth platforms to provide clinical services amid the COVID-19 pandemic?
- **RQ2:** How can telemedicine and eHealth platforms be applied to provide clinical services amid the COVID-19 pandemic?
- **RQ3:** What eHealth software are being utilized to provide clinical services amid the COVID-19 pandemic?
- **RQ4:** Which policies are initiated to foster application of telemedicine to provide clinical services amid the COVID-19 pandemic?

## Findings

### Telemedicine and eHealth software platforms for outpatients

Telemedicine and eHealth platforms refer to the use of computer telecommunications, hardware, and software systems to provide health care remotely or from a distance [14]. It employs real-time interactive visual, textual audio, and data communications to deliver medical-care, consultation, diagnosis, guide, transfer of medical data and treatment. Telemedicine and eHealth platforms are deployed using telephone, Internet Protocol (IP) over Internet voice call, and video discussions [1]. Telemedicine limit exposure to vulnerable patients while simultaneously granting medical practitioners the opportunity to provide care [14]. Additionally, telemedicine can enable outpatients to connect with their physicians at a distance through eHealth platform such as computers or smartphones allowing the physicians to screen patients before they can visit the hospital [15]. This could lead to a significant decline in unnecessary patients visit and encouraging self-quarantine and social distancing [16, 17].

Although, telemedicine can start with telephone consults, other computer technologies such as webcam-enabled personal computers, smartphones, and high speed internet [18], can be employed to provide healthcare to patients [16]. While, face-to-face consultation is undoubtedly important for physician examining patient, it is untenable during pandemics. Thus, researchers such as Leite et al. [19] advocated for outpatients such as those not infected with COVID-19 virus, particularly the high-risk group (pregnant women, older adults with preexisting health conditions, etc.), to use telemedicine as it can provide safe and convenient access to routine treatment without the need to visit health center [20].

Telemedicine can provide appropriate access to routine care without the risk of exposure in a congested health center waiting rooms [20]. Telemedicine and eHealth platforms reduce or eliminates the need for travel for outpatients and delivers cost effective remote services while maintaining quality care [12]. Presently, the adoption of telemedicine and eHealth platforms is presently possible, due to rapid advances in ICT and widespread wireless internet access [13]. Adoption of telemedicine and eHealth platforms is ideal for the management of COVID-19 in slowing the transmission of the virus via social distancing and quarantine thus reducing person-to-person infection [21]. In the current COVID-19 pandemic, the value of telemedicine and eHealth platforms cannot be overstated [12].

### Application of synchronous and asynchronous telemedicine

Telemedicine is adopted either synchronous or asynchronous [13]. Synchronous telemedicine platforms support both patients and physician to establish a real-time video session while exchanging vital data simultaneously. It supports the attending physician to perform remote visual examinations of patient’s condition without essentially having to make direct contact that can expose him/her to the illness being treated [3]. In synchronous telemedicine, patients could consult physicians through eHealth platforms for online health consultation services [17]. Following this initial triage, the physician conducts a remote consultation for further assessment of the risk of COVID-19. Additionally, synchronous teleconferencing can be recorded for professional review and making decisions in regard to treatment of patient and follow ups [1].

**Fig. 1:**
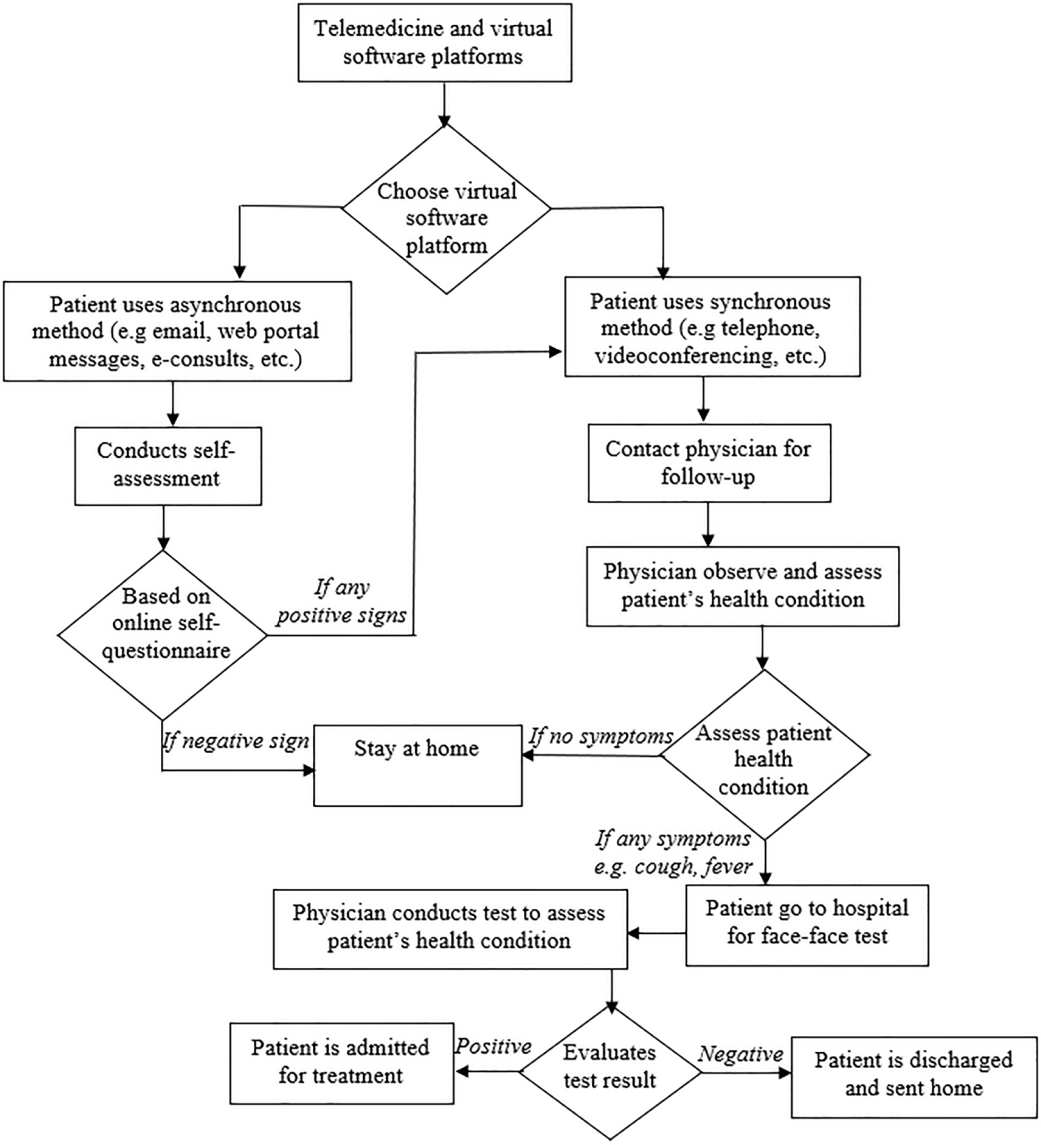
Application of telemedicine and eHealth platforms

Figure 1 shows the application of synchronous and asynchronous telemedicine and eHealth platforms in providing clinical services amid the COVID-19. Furthermore, to reduce exposure and “flattening the curve” of the pandemics telephone and online surveys are employed as important method to prevent outpatients from infection exposure in the first phase of the synchronous teleconsultation [9, 22]. The physician conducts a preliminary screening of patient digitally and also gives suggestions to continue to stay home or to visit the hospital. If patient is to come to the hospital after arrival at the hospital any attending medical practitioner conducts physical test to determine whether patient is suspected for COVID-19 [23].

Patients discharged from the hospital can also be followed up through telemedicine as it provides a medium to seamlessly monitor patients [4]. Asynchronous consultation may be most suitable when adopting in non-urgent case or routine outpatient follow-up [18]. Thus, a patient can send initial medical request and follow-up photos and videos attached to a description of how they are feeling or how they are recovering. After which the attending physician will review the documents [5], and patients will be messaged electronically, or a phone call may be set up or in urgent case in-person visit can be re-schedule [18].

### Types of synchronous and asynchronous platforms used in telemedicine

**Fig. 2:**
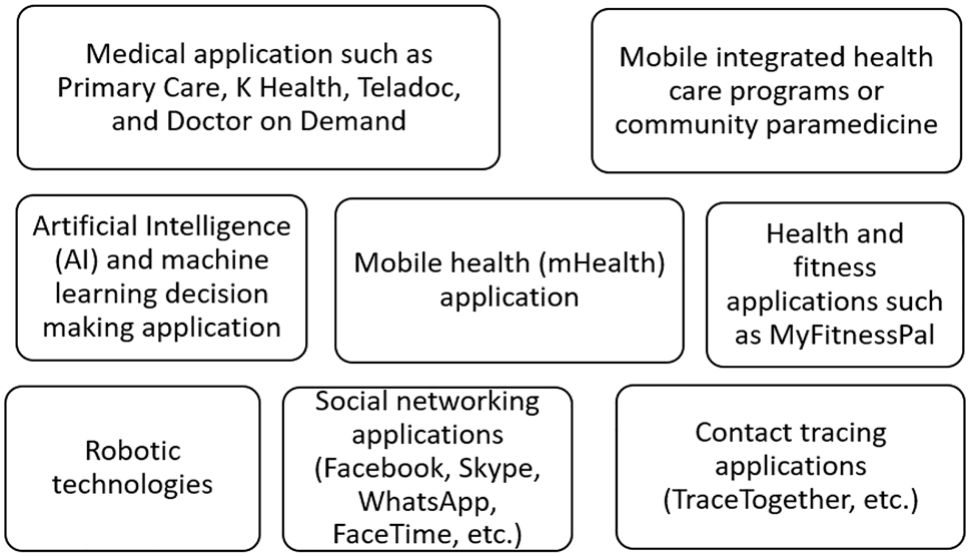
Telemedicine and eHealth platforms currently adopted during the COVID-19

Figure 2 depicts telemedicine and eHealth platforms adopted during the COVID-19 pandemic.

Figure 2 shows the telemedicine and eHealth platforms currently adopted during the COVID-19 pandemic. Each of the software are discussed below;

#### Mobile integrated health care programs or community paramedicine

Mobile integrated health care programs support patients to be treated in their homes, with higher level health support provided digitally oversight by physicians to augment care provider to person who call via emergency channels such as through 911 calls [2], decreasing the need for transportation to the emergency department. This treatment provides virtual emergency consultations and allocate work among subspecialty medical practitioners [2].

#### Mobile Health (mHealth) application

Mobile Health involves the use of mobile device and handheld devices equipped with internet access to manage medical care operations in managing medical data, analyzing medical-related data and improving overall patient experience. The mobile devices install the software application to access their medical information and can be used by physicians to support dissemination of information with other medical practitioners in real-time [1].

#### Artificial Intelligence (AI) and machine learning decision making application

Algorithms are incorporated in telemedicine to assist with conclusive disposition of assessed patients via remote analysis [1, 3]. Thus, AI Chatbot are deployed to provides the latest information on COVID-19 including suggestion on prevention and possible guide to the society. It also provides real-time situation reports to medical practitioners [1]. Presently, AI is being employed to develop COVID-19 screening tools that can be used to conduct preliminary test for patients who have symptoms and further suggest treatment if necessary.

Accordingly, Kaminski [8] argued that AI bots can be deployed to lessen high volumes of patient traffic caused by high calls to health hotlines during this pandemic. Likewise, machine learning models are been used to predict the most likely location of where COVID-19 might be high and can be deployed with AI applications to minimize and expedite the processes involved in diagnoses and monitoring of the infection [24, 25]. Importing COVID-19 infection scanned data to machine learning can help the algorithms learn and improve detection accuracy of the virus. Thus, application of AI and machine learning helps to reduce burden faced by medical practitioners in the current COVID-19 outbreak [24, 26].

#### Robotic technologies

Robotic technologies are being adopted to provide simultaneous and direct support to incapacitated patients faster and also help to provide safety of medical practitioners and volunteers attending to patients affected with COVID-19 [27]. Several robotic technologies used for medical care (e.g. rehabilitation, assistive, and medical robotics), provide support for diagnosis and patient care [1]. With advancement in AI, robots now work faster and serve patients in isolation facility or quarantine center as used in countries such as China for COVID-19 treatment. Moreover, these robots are equipped with in-built mood interpreters to understand patients’ facial expressions, and get feedbacks, assess voice recognition and provide drug administration [1].

#### Social networking applications (Facebook, Skype, WhatsApp, FaceTime, etc.)

Social Networking Applications are being used in self-quarantine and social isolation. Although eHealth platforms cannot replace face-to-face interaction, they provide ease for those who feel lonely and depressed due to the stay at home order/lockdown. These applications can be installed and used in Windows phone, Android, Blackberry, and iOS. It can be installed as desktop software in Windows systems and Apple MacBook among others [25].

#### Contact tracing apps category

Contact tracing is an important method for medical practitioners and municipality administration to manage the spread of COVID-19. Presently, contact tracing applications are being used across the world [28]. For example, the Singaporean government released a mobile application TraceTogether, developed to help health officials in tracking down infected individual and who they may have been in contact with. In Israel, a legislation was passed that permits the government to track the mobile-device data of individuals with suspected infection In Taiwan, health institutions are given access to track phone location data for individuals under quarantine [28].

#### Medical application

Medical applications involve programs that provide both synchronous and asynchronous healthcare care services to patients. eHealth platforms offer medical guide and resources especially to patients residing in undeveloped areas where medical access to care is limited [25]. Besides, eHealth platforms connect patients to remote physicians during natural disaster or emergency when there is increased demand for medical services. Example of medical application software include Primary Care, K Health, Teladoc, and Doctor on Demand, which are all available for download on Google Play and Apple Store. These medical applications provide patients to licensed physicians for non-emergency health problems and are endorsed by the Health Insurance Portability and Accountability Act of 1996 (HIPAA) compliant in US [25].

#### Health and fitness applications

Health and fitness applications are currently used due to quarantine and social isolation which has promote sedentary behavior and decreased physical activity which is a problem for population that spends 60% of the time engaged in sedentary activities [25]. Thus, the use of health and fitness applications could reduce the ill-impact of sedentary behaviors, protect psychological health and help improve sleeping habit. Software such as MyFitnessPal, which provides a diet plan and calorie counter is being used to provide benefit of well-being [25].

### Policies initiated to foster adoption of telemedicine and eHealth platforms

The novel COVID-19 pandemic has considerably changed how medical practitioners treat patients [26]. Also, due to the desire to flatten the curve of transmission, focus is now placed on infection prevention through quarantine and social distancing [13, 27]. Accordingly, to enable patients’ access to medical-care, many countries have revised regulations to allow hospitals and health centers to adopt telemedicine [13, 28, 29]. In the US the Centers for Disease Control and Prevention (CDC) have set out recommendations to prevent infections by reducing or eliminating non-urgent office visits [13, 28]. To expedite adoption of telemedicine in the US, the Stafford Act, enacted in middle of March 2020, permits the Medicare & Medicaid Services (CMS) to extend access for telemedicine services [13, 29], allowing patients to communicate with their physicians through live video conferencing from their homes thereby reducing the risk of exposure and spread of the virus [20].

This has given medical practitioners on the frontlines more flexibility to safely treat outpatients. Consequently, the American Medical Association (AMA) has also developed a new resource for physicians to get advice via telemedicine. It recently launched the AMA telemedicine quick reference guide, aimed at providing best practices for medical practitioners in adopting a broad range of virtual technologies including telemonitoring, telecare, and telemedicine [1]. Also, in US, the State of California in 2019 passed a bill that removes barriers to Medicaid reimbursement for community health centers/clinics amidst states of emergency for telephone services provided from patient’s home [29]. Due to the Covid-19 pandemic the Unites State Congress brought forward, and the President signed the “Coronavirus Preparedness and Response Supplemental Appropriations Act, 2020” which supports the waiving or modifying of Medicare’s telehealth restrictions [26, 29].

China is another country that is actively adopting telemedicine to provide various health services during the outbreak of COVID-19 [27]. eHealth platforms adopted are provided by government and academic organizations to provide psychoeducation, counseling, training, and supervision through eHealth platforms via hotline, Tencent QQ, and WeChat [27]. Thus, telemedicine services have been prioritized for people at higher risk exposure to COVID-19, including patients diagnosed with COVID-19, medical practitioners on the frontline, security guards, and policemen. This was supported by early reports which suggested that people in quarantine actively sought online platforms to address their health needs, which confirmed the society interest and acceptance of telemedicine adoption [27]. Similarly, hospitals in Wuhan, China adopt smart health devices connected to big data analysis systems and remotely monitored and controlled via surveillance cameras from Beijing central administration office enabling medical practitioners to monitor patients' condition without direct exposure. Additionally, the National Telemedicine Centre of China in Zhengzhou also initiated an emergency telemedicine consultation system to remotely manage and monitor the health of patients [1].

In Taiwan, hospitals were provided access to suspected patients travel histories, and health authorities track phone location data for patients under quarantine [28]. Similarly, on March 20^th^, 2020 Singapore released a mobile application (TraceTogether) that tracks via Bluetooth when two application users have been in close proximity to notify of any person reported to be diagnosed with COVID-19 application [28]. The mobile application allows the Ministry of Health to determine citizens login based on a human contact tracer which can be used to call those in contacts with people with the virus to determine appropriate follow-up actions. When the mobile application alerts someone that they have been exposed to COVID-19, the information sent directly from the Ministry of Health. The mobile application helps medical officials in tracking down exposures after an infected person is identified. However, there are important privacy concerns of the existence of the tracking application [28].

In South Korea, the government has manages a public open database of known patients, including information about their gender, age, travel routes, and occupation [28]. The South Korean government utilizes data from social media to aggregate useful telemedical metrics to profile patients’ history in generating automated information to the general public. This helps to shapes the behaviour and cohabitation of residents and provides additional measure against the spread of COVID-19 [1]. Additionally, the European Union (EU) expanded its use of telemedicine to help track and communicate with patients in quarantine and isolation to administer treatments and gather data for evaluation and monitoring of medical outcomes [1]. Based on the Italian cases on COVID-19 infections in accord with corresponding regional method, Italy initiated its telemedicine initiatives which were proactively adopted in 2018 to support greater use of telemedicine and virtual technologies across the country. The adoption of telemedicine proved critically efficient in providing access to medical care services to patients who were otherwise relying on conventional medical-care facilities, some of which were indeterminate to access these services for fear of exposure to COVID-19 [1].

In Israel, a legislation was passed that permit the government to track the mobile phone data of people with suspected COVID-19 infection [28]. One of the Israel’s medical centers has reported the adoption of telemedicine to provide effectively care for 12 Israeli COVID‑19 patients received from the cruise ship that was previously quarantined in Japan. The telemedicine care includes remote patient examination without medical practitioner presence, using robotic telemedicine cart equipped with a screen, camera, and medical equipment controlled by attending physician and nurses, and remotely monitoring using a pulse oximetry, blood pressure instruments, and thermometer, without human presence [3]. The UK’s National Health Service (NHS) introduce the adoption of video consultations by health centers to lessen the number of people who visit the hospitals and decrease the potential for transmission [1, 23].

Additionally, telemedicine services have been previously funded by the Australian Government termed as “Better Access Initiative program” to address health needs of remote and rural patients during emergency circumstances, such as bushfires and long-term drought [20]. To help manage COVID-19, the Australian Government has responded with extra funded services through the Medicare Benefits Schedule which provides greater range of telemedicine services to be delivered [23], including eHealth platforms consultations with medical practitioners and specialists [27]. Evidently, the potential benefits of telemedicine and eHealth platforms are clear, the adoption in emergency situations such as in the current COVID-19 pandemic is still limited across the world.

## Discussion and implications

Currently medical practitioners (medical assistants, nurses, physician assistants, etc.) who work in providing healthcare to patients are adopting telemedicine and ehealth applications [2]. Although, telemedicine has not always been adopted as a viable solution for treating patients because eHealth platforms do not provide key information from virtual examination and diagnostic [25]. Regardless of these barriers, a few medical-care systems in the US such as University of Pittsburgh, Jefferson Health, and Cleveland Clinic [15], have been investing in telemedicine anticipating time when it would become more ubiquitous. Therefore, patients who receive treatment from home can now receive reimbursement [29]. Findings from the literature [11] suggest that US insurers have rapidly expanded medical coverage to include telemedicine and some US states have waived their licensure requirements for care provided beyond state boundaries. Hence, they allow reimbursement of eHealth visits based on United States Department of Health and Human Services (HHS) waived enforcement of HIPAA regulations to allow the use of audio and video communication for telemedicine consultation [11].

Thus, there is need for other medical-centers to transform their health care delivery systems by unleashing the power of eHealth platforms. Although some eHealth platforms, such as those adopted for telemedicine, have existed for years, they have not been fully adopted due to regulation and inadequate funding [26]. Also, adoption of telemedicine in this current pandemic requires medical-centers to abruptly transit to using remote videoconferencing and other virtual solutions, while the medical care system is still managing the COVID-19 crisis. But physicians are faced with limitation when they adopt telemedicine since they require physical examinations of patients such as auscultation and diagnostics which cannot be performed remotely [20]. Although, as stated by Hollander and Carr [2] no telemedicine service can be deployed overnight, hence existing IT systems that have already been implemented can be leverage as response to COVID-19. The basic components for deploying telemedicine system for a medical practitioner comprises of a monitor or computer with an internet-enabled camera and video chat application.

Recent changes in policies across the world have allowed the use of popular social video chat applications such as Zoom, Microsoft Teams, Skype, Google Duo, FaceTime, etc. for patient consultation with a mobile device or tablet [30]. A stable network access and robust IT support are also essential. With the inclusion of extra addons such as digital stethoscope, physicians can perform a digital assessment. Although, this requires dedicated staff training and education in order to optimize the capabilities of telemedicine and eHealth platforms in creating a seamless patient experience [9, 11, 25]. Yet, findings from this study reveal that the adoption of telemedicine has already demonstrated to be an invaluable approach to reduce overwhelming volume of patients from the hospital emergency rooms and transform the work practices of medical practitioners and specialists. As demonstrated by the findings from US, UK, Australia, China, etc. (see section 3.4), well integrated telemedicine and eHealth platforms can reliably manage patients remotely and provide timely care in shortage of medical practitioners [11].

## Conclusion

In this unprecedented time, with practices such as social distancing and self-quarantine in effect toward reducing spread of COVID-19 especially for outpatients’ medical practitioners, and due to lack of medical resources such as PPE. The COVID-19 pandemics has pose challenges to medical-care delivery. Fortunately, we have technology to strengthen our health care system for patients and medical practitioners [26, 31]. Accordingly, it’s time to adopt technological tools such as telemedicine and eHealth platforms into practice. Telemedicine offers an invaluable tool for facilitating timely and safe patient communication and delivery of medical-care services during the COVID-19 pandemic [17, 32]. While limitations exist, explicitly in regard to telemedicine and eHealth platforms capacity to perform complete physical assessment procedures, adoption of telemedicine can substitute or supplement physical treatment [25].

Although adoption of telemedicine and eHealth platforms alone does not guarantee protection against contracting COVID-19, it is useful for frontline medical practitioners. Telemedicine reduce the risks of infection from direct contact for physicians and patients [27]. Findings from this current study present the significance of applying telemedicine and eHealth platforms to provide clinical services amid the COVID-19 pandemic. Besides, the findings present how telemedicine and eHealth platforms can be applied to provide clinical services and also presents eHealth software that are utilized to provide clinical services amid the COVID-19 pandemic. Finally, polices initiated across the world to promote management of COVID-19 are discussed. Future work will examine the factors that impact the application of telemedicine and eHealth in providing clinical services within and beyond the COVID-19 pandemic.
